# Host-Modulation Therapy and Chair-Side Diagnostics in the Treatment of Peri-Implantitis

**DOI:** 10.3390/bios10050044

**Published:** 2020-04-25

**Authors:** Timo Sorsa, Joseph Bacigalupo, Mauno Könönen, Pirjo Pärnänen, Ismo T. Räisänen

**Affiliations:** 1 Department of Oral and Maxillofacial Diseases, Head and Neck Center, University of Helsinki and Helsinki University Hospital, PO Box 63 (Haartmaninkatu 8) FI-00014 Helsinki, Finland; 2 Division of Periodontology, Department of Dental Medicine, Karolinska Institutet, SE-171 77 Stockholm, Sweden

**Keywords:** peri-implantitis, periodontitis, doxycycline, inflammation, matrix metalloproteinase 8, point-of-care testing, treatment outcome

## Abstract

Previous studies report periodontitis and peri-implantitis being able to induce systemic low-grade inflammation, which is known to be associated with increased risk for some systemic medical disease such as cardiovascular disease. In this regard, recent studies have shown that host modulation therapy (HMT) together with traditional mechanical and surgical treatment not only cease the progression of periodontitis but also reduce the systemic collagenolytic biomarkers in both oral fluids and circulation. This suggests that the corresponding adjunctive HMT-medication could be effective in the prevention and treatment of dental peri-implantitis, as well. Furthermore, low-cost, safe, and practical oral fluid active matrix metalloproteinase-8 (aMMP-8) lateral-flow immunotests have been proposed as point-of-care/chair-side diagnostic tools to detect peri-implantitis and periodontitis, and to monitor their effective resolutions, while using various therapeutic strategies, including host modulation. This study reports the potential benefits of HMT-medication in the prevention and treatment of dental peri-implantitis among five patients (four of five were current/ex-smokers). In addition, the aMMP-8 point-of-care test diagnosed 20 peri-implantitis and 20 healthy controls correctly. In conclusion, this study and previous studies support the potential effectiveness of HMT-medication(s) and point-of-care/chair-side technologies in the treatment and diagnostics/monitoring of peri-implantitis. However, more studies are needed to further confirm this.

Dental implants have become a critical strategy to improve oral function and esthetics in partially and completely edentulous patients [1]. Increasingly, this treatment strategy has involved not only medically healthy patients but also patients with systemic diseases such as diabetes, cardiovascular and gastrointestinal diseases, and others [1,2,3]. Although in many cases implant treatments have been described to be successful in medically compromised patients, “there is insufficient evidence to conclude whether dental implants can remain functionally stable option” in these patients [1]. Many studies have evaluated the success of dental implant treatments [1], but much less attention has been paid to the risks, if any, related to proinflammatory peri-mucositis and peri-implantitis and their potential to induce systemic low-grade inflammation. These two peri-implant diseases affect 19–65% of patients and may have a negative impact on their health, as systemic low-grade inflammation is known to be associated with increased risk for cardiovascular and other systemic medical diseases [4,5].

In this study, we now propose that the potential complications of peri-implant disease require more intense treatment, not only the common disinfection and management protocols for the local oral lesions but also all the systemically-administered host-modulation therapies (HMT) that are currently, and in the future, available [1,4,5]. This proposed “combination” therapy not only enhances the efficacy of conventional dental treatments/managements for peri-implantitis but also reduces the risks for systemic inflammation and disease(s) [4,5,6]. In this regard, this short communication further studies and assesses the potential benefits of administration of HMT to the treatment of dental implant patients (Table 1, Figure 1), and also the benefits of active matrix metalloproteinase-8 (aMMP-8)-based oral fluid point-of-care/chair-side diagnostics (Figure 2, Figure 3) to the peri-implant disease diagnostics/monitoring. Previously, HMT administration to patients with periodontal diseases has been studied among postmenopausal osteopenic women [5,6], and others such as patients with cardiovascular disease [7,8,9,10,11,12,13,14]. It should be also noted that, in addition to MMP-8 (Figure 2), there are also available other well-established “biomarkers” of both tissue destruction and systemic inflammation, such as MMP-9, high-sensitivity C-reactive protein (hs-CRP), TNF-α, and IL-6, which are readily available and detectable in both oral fluids and blood samples [6,7,8,9,10,11,12,13,14,15]. Additionally, other potential biomarkers and test technologies currently exist and are under intensive research [16]. These may be useful, as well, and should be evaluated in the future studies.

Periodontitis and peri-implantitis-related systemic low-grade inflammation can thus also be readily monitored in oral fluids and serum samples, by measuring biomarkers, including MMP-8 and -9, pro-inflammatory mediators (TNF-α, IL-6), acute phase proteinases, and CRP, to diagnostically reveal the risk (Figure 1 and Figure 2) [6,7,8,9,10,11,12,13,14,15,16]. These biomarker measurements are indeed suitable for patients at evident cardiovascular risk to assess requirements for therapeutic interventions and medications but also for monitoring metalloproteinase inhibition with sub-antimicrobial doses of doxycycline to prevent acute coronary syndromes (MIDAS) and reduce harmful systemic low-grade inflammation [6,7,8,9,10,11,12,13,14,15]. In this way, biomarkers’ usage in monitoring diseases and their treatment have become clinically practical and fruitful with the current availability of point-of-care (PoC)/chair-side biomarker analysis of oral fluids (Figure 1, Figure 2, Figure 3) and serum [7,8,9,10]; host-modulation medications, including non-anti-microbial doxycycline regimen (Periostat^®^, now generic; and Oracea^®^), as pleiotropic MMP-inhibitors; and others, including omega-3 fatty acid derivatives (e.g., docosahexaenoic acid), i.e., resolvins [5,6]. Thus, recently-developed strategies of personalized use of HMT (Table 1), when monitored by the modern PoC/chair-side diagnostic aMMP-8-tests (Figure 1, Figure 2), may significantly enhance the beneficial results of both the local oral therapy (scaling and root planing, and oral hygiene instruction) and its effects on the systemic medical health of the patients (Table 1, Figure 1) [6,7,8,9,10,11,12,13,14,15]. Additional evidence of the need for modern, biologically based diagnostics and therapeutics to manage the oral/systemic health of patient is consistently increasing [5]. Such “two-pronged strategies” are at present available to the dental clinician as the result of long-term basic and translational research [5,6,7,8,9,10,11,12,13,14,15].

The diagnostics used for periodontal and peri-implant diseases has mainly relied on the clinical measurements of pocket depths, attachment loss, and bleeding on probing (BOP), together with x-ray analysis [1,4,5,7,8,9,10]. However, such diagnostic procedures can evaluate mainly past tissue destruction and thus do not produce any sufficient information about the ongoing disease activities and/or potential future risk of disease progression. Notably, the periodontal and peri-implant probing and BOP assessment always cause bacteremia, whereas noninvasive PoC/chair-side aMMP-8 oral fluid tests (Figure 2) never cause bacteremia and are always sterile [7,8,9,10]. Moreover, previous studies and the ROC analysis in Figure 3 show that PoC/chair-side aMMP-8 oral fluid tests screen on-line the tailored dental/oral/implant health, disease, and hygiene significantly more exactly than BOP [7,8,9,10]. Therefore, there is a clear need for the use of biomarkers to screen/diagnose sites and patients prone to these diseases in order to do a well-timed intervention and potentially prevent peri-implant and periodontal tissue destruction [7,8,9,10]. Collagenase-2/neutrophil-derived collagenase (also known as MMP-8) and especially its active/activated forms [7,8], have recently been identified as major collagenolytic protease, causing irreversible soft and hard tissue destruction during peri-implantitis and periodontitis [7,8,9,10]. In this regard, successful research has been performed in peri-implantology and periodontology by developing the quantitative PoC/chair-side lateral flow immunoassays for active MMP-8 (aMMP-8) in oral fluids (saliva, mouth rinse, gingival crevicular fluid [GCF], and peri-implant sulcular fluid [PISF]) as diagnostic dental peri-implant and periodontal disease biomarkers (Figure 2, Figure 3). Therefore, the low-cost, safe, and practical oral fluid aMMP-8 lateral flow immunotests have been proposed as a practical PoC/chair-side diagnostic tools resembling classical pregnancy tests (Figure 2) to alarm and detect early peri-implantitis and periodontitis before clinical and x-ray manifestations [7,8,9,10], and to monitor their effective resolutions using various therapeutic strategies including host-modulation (Table 1, Figure 1, Figure 3).

Notably, our group also repeatedly demonstrated that treating these patients with HMT (i.e., non-antibiotic-dose doxycycline as a pleiotropic MMP-inhibitor) not only reduced the severity of chronic periodontitis but also decreased the biomarkers of systemic inflammation (hs-CRP, TNF-α and IL-6) and collagenolysis (MMP-8 and -9) in the circulation as well [5,6,11,12,13,14]. With this background and to conclude, we now propose that animal as well as human studies be conducted to further determine whether HMT, as an adjunct to the traditional dental implant therapies [5], reduces the incidence and severity of peri-implantitis locally, while at the same time addressing the potential ability of HMT to reduce the systemic inflammation (and related biomarkers levels), and, thereby, the eventual risk for related medical conditions, including cardiovascular disease, diabetes, and stroke [5,6,7,8,9,10,11,12,13,14,15]. Furthermore, there exists, and are also under development, some new and recent promising methods, e.g., a safe, natural-product-derived anti-inflammatory and antimicrobial lingonberry mouth rinse treatment/intervention that can be used for health promotion and disease, inflammation, and infection prevention in the oral cavity [18]. Such management and interventions may prove to be beneficial in the treatment of peri-implantitis and periodontitis, but more future studies are needed for further conclusions.

## Figures and Tables

**Figure 1:**
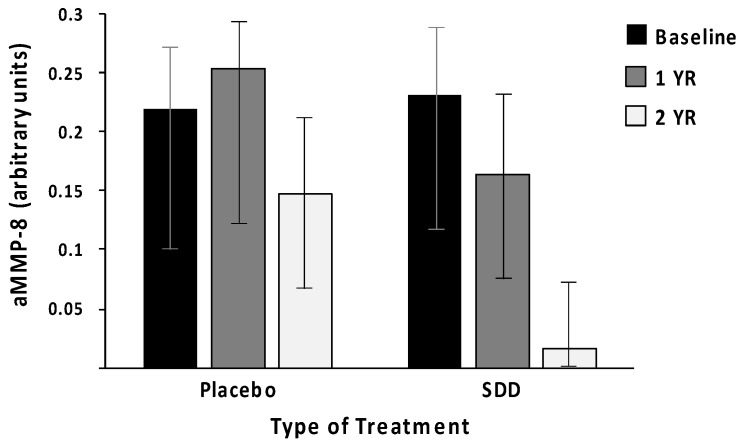
Subantimicrobial-dose doxycycline (SDD) administration reduces the risk of high levels of active MMP-8 (aMMP-8, neutrophil-type collagenase) in periodontal pockets (GCF) of post-menopausal women with chronic periodontitis, during a 2-year double-blind placebo-controlled study (n = 128 subjects). Based on both “intent-to-treat” and on “per-protocol” statistical analyses, the odds of high aMMP-8 were significantly reduced by 60 % (p = 0.006) and 78 % (p = 0.007), respectively, by SDD treatment (logistic regression analysis; bar plot with error bar (95% confidence interval) labeled, data modified from Golub et al. [17]). Based on the recent periodontitis classification of Tonetti et al. [15], the patients with grade C (i.e., severe-progressive periodontitis) prior to placebo (only standard treatment) or SDD treatment were reduced to grade A, i.e., minimally-progressive disease. Measurements of aMMP-8 levels are done by Western blot (arbitrary units) (Golub et al. [17]).

**Figure 2:**
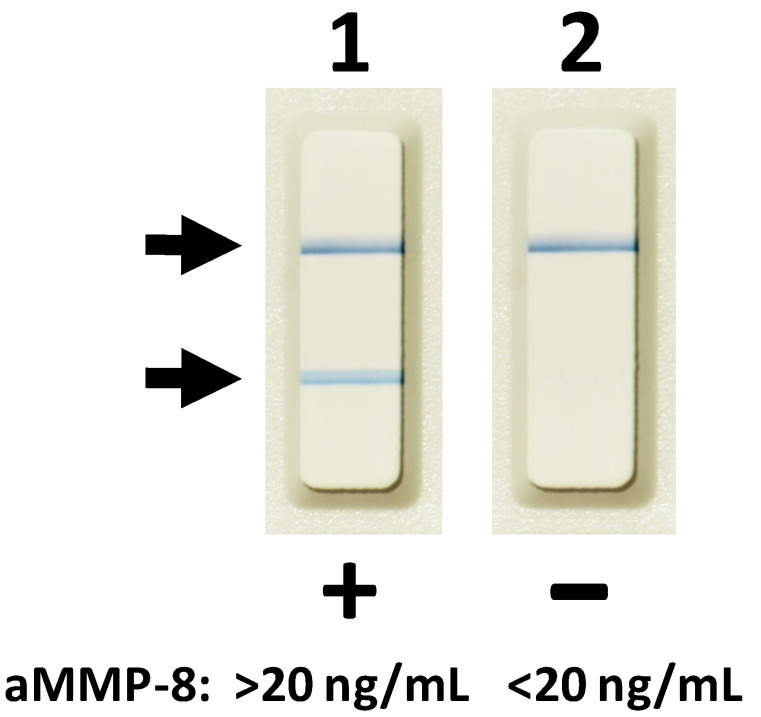
Oral fluid (saliva, mouth rinse, gingival crevicular fluid (GCF), and peri-implant sulcular fluid (PISF)) lateral flow aMMP-8 point-of-care/chair-side immunoassay. Lane 1, two lines indicate aMMP-8 levels >20 ng/mL in GCF and a risk of progressive and active peri-implantitis before 3-month subantimicrobial-dose doxycycline (SDD) medication as adjunctive to scaling and root planing. Lane 2, one line indicates aMMP-8 levels <20 ng/mL in GCF and a reduced risk of progressive and active peri-implantitis at 3 months after scaling and root planing treatment and adjunctive SDD.

**Figure 3:**
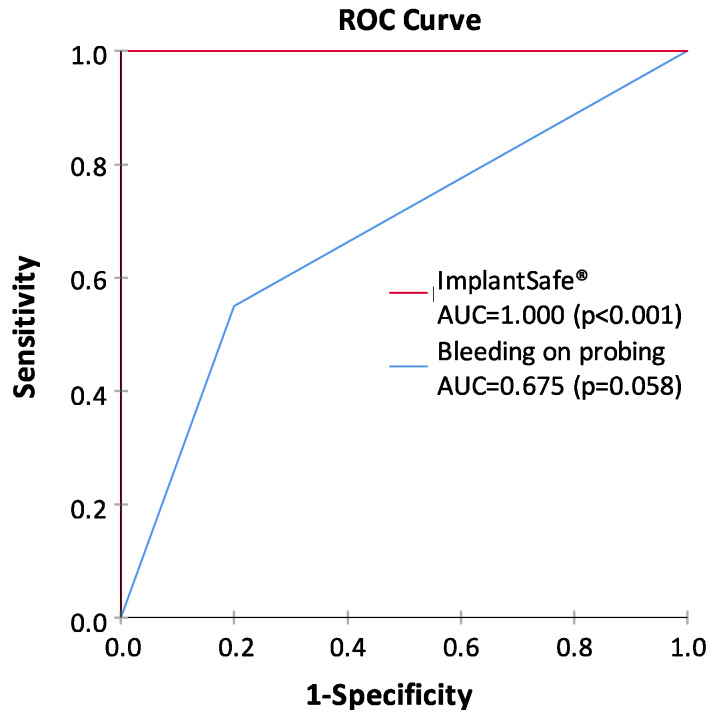
Receiver operating characteristic (ROC) curves and the area under the ROC curve (AUC) values for the ImplantSafe^®^/aMMP-8 point-of-care test and bleeding on probing (at least one site bleeding around dental implant), representing the ability of both tests to classify peri-implantitis and health. Peri-implantitis patients (n = 20) and healthy controls (n = 20) were characterized clinically and from X-rays as described earlier [10]. The study was conducted in accordance with the Declaration of Helsinki; participants provided written, informed consent; and the protocol was approved by the local ethical committee of Stockholm Community, Sweden (2016-08-24/2016/1:8 and 2016-1-24; Dnr 2016/1410-31/1) and the Helsinki University Central Hospital, Finland (2019-6-26; Dnr HUS/1271/2019).

**Table 1:** Prevention and/or treatment of peri-implantitis by oral (systemic) host-modulation therapy (subantimicrobial-dose doxycycline, SDD).

Patient No.	Age/Sex	No. of Implants/Duration	Duration of Periostat^®^	Periostat^®^		Current P.I. Status
				Prevented P.I.	Reduced P.I.	(Clinical + x-Ray Evidence)
Case #1	73/M	5/9 years *	6 years		√	None
Case #2	50/F	1/12 years *	4 years	√		None
Case #3	84/F	2/19 years *	17 years	√		None
Case #4	65/F	4/19 years *	3 years		√	None
Case #5	62/M	1/11 years	3 years	√		None

Periostat^®^: Non-anti-microbial doxycycline medication: P.I.: peri-implantitis; M: male; F: female; *: current or previous smoker. Five healthy patients (mean age = 66.8 years) with 13 implants in place for a mean of 14 years were treated with SDD for an average of 6.6 years (J.B.). All five patients currently show no clinical or x-ray evidence of P.I. (note that four of five P.I. patients are or were smokers*, and all are elderly, and both issues are risk-factors). In conclusion, long-term administration of HMT (SDD) (3–17 years), adjunctive to local disinfection, may effectively prevent and treat peri-implantitis. The study was conducted in accordance with the Declaration of Helsinki; participants provided written informed consent, and the protocol was approved by the local ethical committee of Stockholm Community, Sweden (2016-08-24/2016/1:8 and 2016-1-24; Dnr 2016/1410-31/1) and the Helsinki University Central Hospital, Finland (2019-6-26; Dnr HUS/1271/2019).
